# Efficacy of novel antiseptic product containing essential oil of *Lippia origanoides* to reduce intramammary infections in cows

**DOI:** 10.14202/vetworld.2020.2452-2458

**Published:** 2020-11-17

**Authors:** Natalia Arantes Marcelo, Viviane Aguiar Andrade, Cintya Neves Souza, Rodrigo Pereira Mourão, Mário Henrique França Mourthe, Lívia Mara Vitorino Silva, Alessandra Rejane Ericsson de Oliveira Xavier, Mauro Aparecido de Sousa Xavier, André Augusto Gomes Faraco, Anna Christina Almeida

**Affiliations:** 1 Institute of Agricultural Sciences, Federal University of Minas Gerais - ICA/UFMG, Campus. Montes Claros, Minas Gerais, Brazil; 2 Department of Physiopathology, Biological Sciences and Health Center, State University of Montes Claros - UNIMONTES, Montes Claros, Minas Gerais, Brazil; 3 Pharmaceutical Science Post-Graduation Program, Faculty of Pharmacy, Federal University of Minas Gerais, Belo Horizonte, Minas Gerais, Brazil

**Keywords:** Intramammary infections, *Lippia origanoides*, mastitis, pre- and post-milking, teat disinfectant

## Abstract

**Background and Aim:**

The use of antimicrobials in the control of mastitis is of concern in public health due to their inefficiency in targeting microorganisms. Studies with medicinal plants have risen as an alternative to the use of conventional products. The objective of this study was to evaluate the efficacy of an experimental disinfectant based on the essential oil (EO) from *Lippia origanoides* in preventing the development of new intramammary infections (IMI) in Holstein cows.

**Materials and Methods:**

The conventional protocol of pre- and post-milking was used and the control (Conventional treatment [CNV]) and experimental (Experimental treatment [PEX]) products containing EO at 120 μL/mL were applied by immersion. Individual milk samples were analyzed using sheep blood agar methodologies and biochemical tests. The efficiency of the treatment was defined by the presence or absence of *Staphylococcus aureus*, coagulase-negative *Staphylococcus*, and *Streptococcus* spp.

**Results:**

There were no clinical and subclinical mastitis cases, no lesions in the mucosal of teats, nor dirt score between groups in this study. Both treatments did not influence the occurrence of IMI.

**Conclusion:**

The results revealed that PEX acts efficiently against microorganisms compared to the disinfection by the conventional product demonstrating the efficacy of the alternative product on the prevention of new IMIs in dairy cows.

## Introduction

Mastitis is a major economic problem for the dairy industry and the predominant causes of bovine mastitis are intramammary infections (IMI) with bacteria [1]. Expenses with respect to the prevention or treatment of mastitis and losses due to low milk production or disposal of milk directly influence the revenue and profitability in dairy herds [1,2]. Research has shown that the expenses of preventive treatment corresponded to a maximum of 13.5% of the economic impact [3]. This percentage was lower than the economic impact of the expenses of curative treatment. Therefore, the control of mastitis on the property is an important factor in reducing milk contamination.

*Staphylococcu*s and *Streptococcu*s bacteria alter the centesimal composition, somatic cell number (somatic cell counts), and the microbiological, physical, and chemical qualities of milk [4]. *Staphylococcus* spp. is mainly found in milk samples from animals with clinical mastitis (CM) [5] *w*hile *Staphylococcus* coagulase-negative (SCNs) are frequently isolated in bovine milk and are considered similar to secondary pathogens [6] that promote the inflammation of the mammary gland [7].

The utilization of antibacterial compounds in the sanitization of teats has been viewed with great concern by the public health and alimentary security. Hence, different methodologies are being explored to prevent the development of mastitis in the herds, including the development of vaccines. A recent study of vaccines in the prevention of *Staphylococcu*s *aureus* bovine mastitis suggests that live-attenuated SCVs vaccines can induce strong humoral and cell-mediated immune responses, which are crucial for protection [8]. As the use of vaccines is still not well established and there is an indiscriminate utilization of antibiotics to control mastitis, the research on the development of alternative therapeutic methods is heightening [9].

Some research seeks to find natural products with antimicrobial activities to be used in animal production and in the control of bovine mastitis. Medicinal plants and plant-derived essential oils (EO) have active biologics with bactericidal characteristics that are of significance to animal production since the proven inhibitory and antiseptic activity [10]. In addition, they are potential alternatives to conventional medicines that may cause resistance [10].

*Lippia origanoides*, whose EO is extracted from its leaves, is a shrub native to the semi-arid regions of Minas Gerais, and it has antimicrobial activity [10]. Chromatography combined with gas chromatography-mass spectrometry (CG-EM) has been used to elucidate the chemical composition of the EO of *L. origanoides*. The most important constituents identified were carvacrol (ranging from 29% to 32%), followed by o-cymene (23% to 25.57%) and methyl thymol ether (10.03% to 11.50%) [10,11]. These compounds have shown antimicrobial activity against *S. aureus* and *Streptococcus mutans* [10-12], the most prevalent microorganisms in mastitis. A concentration of 120 μL/mL of *L. origanoides* EO inhibited the growth of *Escherichia coli*, *S. aureus*, and *Salmonella choleraesuis* without causing cellular toxicity [10]. At the same concentration, the EO presented antiseptic activity against these microorganisms and inhibited their growth after 5 min (for *S. aureus*) or 15 min (for *S. choleraesuis*) of treatment [11]. These studies suggest that the *L. origanoides* EO can be used to formulate antiseptic products capable of slowing microbial growth.

The aim of this study was to verify the efficacy of an alternative antiseptic product, containing the EO of *L. origanoides*, and its usability in the sanitization in pre- and post-milking disinfection of teats to reduce the microorganisms in bovine milk and to control IMI.

## Materials and Methods

### Ethical approval

This research was performed within the ethical standards approved by the Animal Use Ethics Committee (CEUA) of the Federal University of Minas Gerais (UFMG) under protocol number 230/2014.

### Animals test

A total of 16 Holstein cows (64 functional teats without CM) of lactation duration ranging from 100 to 200 days (DEL) obtained from the Hamilton de Abreu Navarro Experimental Farm (FEHAN) located at the Campus of the Federal University of Minas Gerais (UFMG), in Montes Claros-MG, were enrolled in this trial from October to December 2015.

The diagnostic tests for clinical and subclinical mastitis (SCM) were performed twice a day for 1 month, from the commencement of the experiment and until the end of the experiment. The diagnosis of CM in cows was determined by the examination of changes in the udder, detection of flakes and clots with gland swelling or redness, rise in temperature, swelling, hardness of udder, and changes in milk color, or systemic illnesses such as fever, depression, weakness, and dehydration. For the diagnosis of SCM, the California Mastitis Test was used as reported. The milking equipment and management practices were monitored once a day to minimize machine-mediated infections.

Each test group was composed of 32 teats. The teats eligible for inclusion in the trial were defined by the absence of *S. aureus*, SCN, and *Streptococcus* spp., defined as indicators of infectious agents. Initially, the defined bacteriological status was that each teat, in the first milk gathering of the single-sample treatment period, did not show indicators of infectious agents. Duplicate milk samples were collected from each teat to determine the bacteriological status of the quarters within 7 days before the initiation of the trial [13].

### Treatments administered

The two treatments which were administered were the experimental teat disinfectant (PEX) and conventional disinfectant (CNV). Each treatment was administered to two teats in each udder and was distributed in such a manner that a different pattern of quarters was allotted the two treatments in each cow to account for any dependency among quarter location with respect to the incidence of mastitis [13,14].

The conventional protocol of pre- and post-milking (CNV) was adopted; chlorhexidine 1% (Hexiderm^®^, Brazil) and iodine 2500 p.p.m. (ULTRADIP 2500^®^, Brazil). The experimental product (PEX) was prepared with 120 μL/mL of EO from *L. origanoides* provided by the UFMG School of Pharmacy. The PEX product was tested and answered all the requirements for veterinary antimicrobial products as recommended by the Brazil regulatory affairs [15]. The CNV and PEX products were applied by immersion during pre- and post-milking without the return of the applicator for reuse. The storage was verified and maintained in accordance with the manufacturer’s recommendations, ensuring proper application in the treatments [5].

The score of mucosal integrity was analyzed (EIET < 3) in addition to the dirt score (EST < 4) [16-18]. The cows were milked twice a day at 6:00 am and 2:30 pm. It is notable that the teat scoring should take place before starting the experimental exposure and at least every 6 weeks during the trial. For the identification of CM, milkers were trained to identify any abnormal milk or clinical symptoms associated with a CM case. To confirm CM, milkers discarded the first streams of milk in a strip cup before applying the pre-milking teat disinfectant. If a CM quarter was identified, milkers collected the milk aseptically before mastitis treatment, and information from each CM episode was recorded [19].

### Study design

Quarter foremilk samples were collected weekly during the trial. All samples were collected immediately before regular milking. The milk collection followed the methodology described by Simões *et al*. [20] and the samples were sent to the Animal Health Laboratory located at the Institute of Agricultural Sciences of UFMG. Subsequently, the microorganisms were isolated and identified following Brito’s methodology [21] and the microbiological identification of the isolates was performed [20-22].

The efficiency of PEX was evaluated following the criteria indicated by Schukken *et al*. [13], Ceballos–Marquez *et al*. [5], and the National Mastitis Council [23]. A new IMI in a quarter was diagnosed when the same bacterial species is isolated from two out of three consecutive samples during the trial. An individual quarter is eligible for only one new IMI per bacterial species during the trial. In case of the occurrence of CM, the animal was excluded from the study [5,24]. A complete cure was determined when the samples presented without pathogens in three consecutive samplings [16].

The evaluation of the effectiveness of PEX was performed from the rates of reduction of effectiveness observed during the trial period when compared to the control [5], according to the equation: Rate of IMI with PEX/Rate of IMI with control product. The recommendations of Ceballos–Marquez *et al*. [5] were used as criteria to compare the product to the control [5]; a margin of no inferiority was necessary (d=50%) [23]. It was defined that:

(1) H_0_= Rate of IMI with PEX—rate of IMI with CNV (≥d, PEX is inferior to CNV).

(2) HA = Rate of IMI with PEX—rate of IMI with CNV (<d, PEX no inferior to CNV).

### Statistical analysis

The frequency and occurrence of *Staphylococcus* spp. and *Streptococcus* spp. were evaluated using the two treatments in pre- and post-milking. The difference observed in the rate of IMI between treatments was calculated by the Qui-square test. The hypothesis used was that there were no significant differences between CNV and PEX pre- and post-milking in the reduction of natural infections [5]. The GraphPad Prism, Version 5 (GraphPad Software, USA) [25] was used for a comparative analysis between the standard media of IMI. Pearson’s correlation evaluated the positive or negative correlation between the bacterial presence in both treatments.

## Results and Discussion

The present study did not record any cases of observed clinical and SCM cases, lesions in the mucosal of teats, nor dirt score, as related previously [26]. These results indicate that the disinfectant agents used in this study did not interfere in the incidence of new IMI. It is known that one of the desirable characteristics of a disinfectant is its non-toxicity or lack of irritating properties in addition to the promotion of good hygiene and maintenance of the integrity of teats throughout the treatment. It is also important to follow-up on the presence of organic matter in the skin of teats as the washing of the teat before milking is considered a protective factor for mastitis [27]. Hence, a teat disinfectant must have several characteristics: Germicidal efficacy, ability to prevent new IMI, ability to maintain teat condition, and no deposition of residues in milk that can affect human health [28].

Therefore, the determination of the efficacy of teat disinfectant products against bacteria naturally present on the teat skin is important. A study determined the impact of ten pre- and post-milking teat disinfectant products with different ingredients of varying concentrations on the reduction of teat skin bacterial load without damaging the teats [29]. The results revealed that all the tested teat disinfectant products reduced the teat bacterial load for all three bacterial groups [26]. Of these, it was notable that the product containing 0.6% w/w diamine was the most effective against the bacterial populations of staphylococcal and streptococcal isolates on teat skin with a reduction of 90% and 94%, respectively, while another product containing 0.5% w/w iodine resulted in the highest reduction (91%) in coliforms on teat skin [29]. The results from this study suggest that specific bacterial population loads on teats can be reduced using different teat disinfectant formulations. The results of this study corroborate with the previous results, as the product disinfectants tested here did not cause any alteration in the incidence of new IMI.

**Figure-1:**
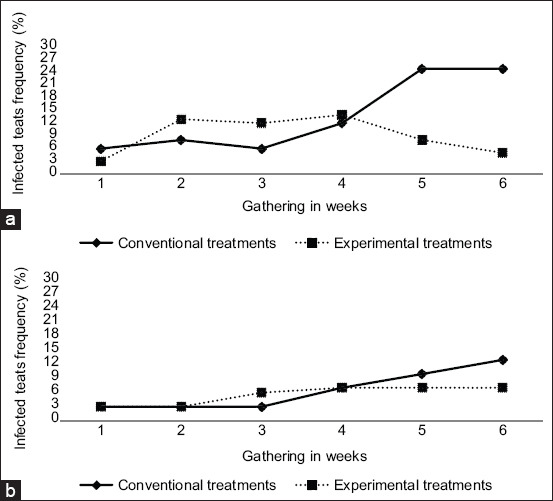
Frequency of coagulase-negative (a) *Staphylococcus* and (b) *Streptococcus* spp. isolates in milk samples of Holstein cows subjected to two treatment of sanitization of teats for 6 consecutive weeks.

On investigation of the presence of the main pathogens that cause mastitis, it is notable that *S. aureus* was not found in any of the 384 samples of milk. It was noticed that 13.54% of microorganisms belong to SCN (n=26/192) and 9.89% to *Streptococcus* spp. (n=19/192) isolated during treatment. The good practices in milking and appropriate management, as well as the separation of animals in batches during the experimental period may have contributed to the absence of pathogens. Hence, this indicates that the tested products, both control and treatment, were efficient in preventing microorganisms from entering by the ceiling channel causing mastitis. This can explain the absence of *S. aureus* in the experimental animals. It is notable that no significant difference was observed between the sampling and the treatments for both SCN and *Streptococcus* spp. during the trial (p>0.05) (Figure-1).

**Figure-2:**
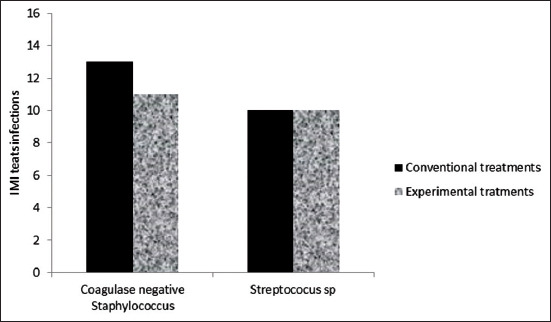
Coagulase-negative *Staphylococcus* and *Streptococcus* spp. isolates in milk samples from infected teats sanitized with conventional (CNV) or experimental product (PEX) with *Lippia origanoides* essential oil.

The presence of microorganisms in the groups of teats was identified by comparison between the teat groups. No significant difference was observed in the frequency of SNC and *Streptococcus* spp. (p>0.05) (Figure-2). Although no significant difference was observed between the used products, it is important to consider the frequency of SNC and *Streptococcus* spp. observed in the results of Figure-1 as these microorganisms are potentially pathogenic. Mello *et al*. [30] established similar results and described that the presence of these pathogens characterized the occurrence of contagious mastitis, indicating that prophylactic measures and the adoption of pre- and post-milking teat disinfection are essential in mastitis control. Sampimon *et al*. [6] considered SNC as secondary pathogens with a large prevalence in cattle herds. It can cause moderate mammary infection acting such as a causative agent of clinical and SCM with severe disease [27], increased conventional CS [4], and reducing the milk production [16].

The residual milk promotes the development of SNC by acting like a culture medium leading to IMI [16]. Various results indicate the relevance of SNC in the occurrence of clinical and SCM in Brazilian cattle with a frequency of 8.35% of CM [27] and varying from 4.8 to 8.35% in SCM [7,27]. Therefore, a high prevalence in the herds can be associated with a deficiency of teat disinfection practices in the pre- and post-milking routines [16].

A new product for the disinfection of teats based on copper and zinc (experimental teat disinfectant ZkinCu) was tested in a herd with an automatic milking mechanism to verify new intramammary cases under natural infections. The results of the study showed that the experimental teat disinfectant ZkinCu, evaluated in this field trial with naturally occurring IMI, showed non-inferiority relative to the positive control for the prevention of new IMI [28]. The predominant organisms recovered from the quarters with new IMI were *Streptococcus uberis*, *Corynebacterium* spp., and coagulase-negative staphylococci in both the ZkinCu and OceanBlu groups. It was observed that the risk of infection in the OceanBlu group was higher (β=0.644; 95% confidence interval=0.05.1.22). The interaction of treatment by week was not significant. The new IMI rate estimates (95% confidence interval) for ZkinCu and OceanBlu were 1.7% (0.8.2.5) and 3.2% (1.7.4.7), respectively [28].

These results suggest that the low frequency of major pathogens such as *S. aureus* could be due to the well-managed sanitation of the herd in relation to udder health. This finding corroborates with the results of our studies to emphasize the importance in applying good practices of teat management and sanitation in pre- and post-milking associated with a good disinfectant product that does not cause damage to the teat.

**Figure-3:**
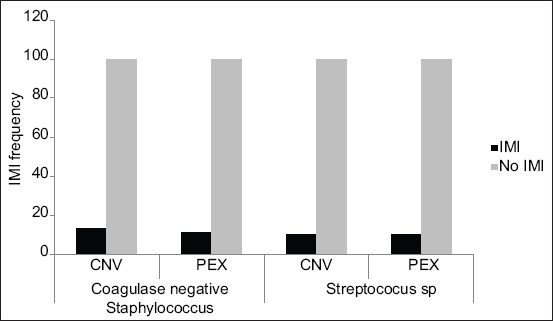
Frequency of new intra-mammary infections (IMIs) caused by coagulase-negative *Staphylococcus* or *Streptococcus* spp. observed in teats treated conventional (CNV) or experimental product (PEX) containing *Lippia origanoides* essential.

The Pearson’s correlation between the presence of isolated agents in the different treatments was negative (−0.031), indicating that there was no correlation between these analyzed parameters. Quirk *et al*. [16] evaluated the effectiveness of the iodized solution after dipping and observed that there was a reduction in the infection-causing species in quarters treated with iodine. The results presented here show that the occurrence of infected teats was statistically similar (p>0.05) for both PEX and CNV protocols (Figure-3).

With these encouraging results, the rates of IMI induced by SNC and *Streptococcus* spp. were evaluated; additionally, no significant difference was observed (p=0.8884), as this value was smaller than the PROB value (0.9711). This suggests that the conventional and alternative treatments did not interfere with the occurrence of IMI by the microorganisms under study. The efficacy of PEX analyzed in this study showed a rate of 0.41 for new infections by SCN, while *Streptococcus* spp. showed a rate of approximately zero (0) (Figure-3). This value is according to the recommendations of Ceballos–Marquez *et al*. [5], in whose work; the value of 0.5 was adopted. There was no significant difference (p>0.05) for SCN and *Streptococcus* spp. regarding IMI in both treatments (Figure-3). When assessing the non-inferiority margin (d=50%) [31], it is observed that the effectiveness of the PEX is not inferior to the CNV.

There is a preoccupation about the presence of iodine residues in milk and its accumulation in animal tissues and a crescent resistance of microorganisms to conventional products [14]. Therefore, the use of plant extracts in the sanitization of equipment and facilities, treatment, and disease control of farm animals has been authorized by the Normative Instruction (IN) Number # 64 of September 18, 2008, from the Ministry of Agriculture, Livestock, and Supply — MAPA [32].

Current research is analyzing alternative control methods and medicinal plants that contribute to natural, safe, effective, and inexpensive options. Studies on *Minthostachys verticillata* demonstrated that the EO of this species and limonene, one of its compounds, inhibited the growth of mastitis pathogens [33]. The EO of *M. verticillata* with a minimal inhibitory concentration of 29.0 mg/mL was more effective than limonene, also showing bactericidal action against *Enterococcus faecium* [33]. This oil affected the mature biofilm of isolated strains serving as a therapy against bovine mastitis pathogens by inhibiting the development of pathogenic bacteria [33].

The antimicrobial activity of crude ethanolic extract from the bark and leaves of *Commiphora leptophloeos* against the isolates of *Staphylococcus* spp. from the milk of ruminants with SCM was evaluated. The extracts of the bark and leaves at a concentration of 781.2 μg/mL were able to interfere with the initial stages of biofilm formation; however, there was no interference of the extract on the established biofilm [34]. There was a high sensitivity of *Staphylococcus* spp. isolates from SCM cases in ruminants when subjected to the extracts from the bark and leaves of *C. leptophloeos*, with respect to the ability of extracts to interfere in biofilm formation, indicating their potential in use for ruminant mastitis therapy [34].

Oleoresin (OR) and the EO of *Copaifera* spp. were investigated against microorganisms isolated from milk samples of cows diagnosed with Grade III SCM. The OR exhibited significant antimicrobial activity (minimum inhibitory concentrations [MIC] ≤100 μg/mL) against samples of *Staphylococcus* coagulase-positive, *Staphylococcus* coagulase-negative, and *Streptococcus* but were inactive and weak to inactive, respectively, against *E. coli*. This demonstrates that this raw plant material is promising for the development of phytotherapeutic drugs against bovine mastitis [35].

Sperandio *et al*. [36] investigated the antimicrobial activity and the cytotoxicity of *Tagetes minuta* L. EO against *S. aureus* and *E. coli* and observed a MIC of 1.0 mg/mL for *strain of S. aureus* and five bacteria isolated from mastitis milk, including a multi-resistant strain; and 3.0 mg/mLfor strain of *E. coli* and two bacteria isolated from mastitis milk. However, a strong cytotoxic effect in the MAC-T cells was found in the oil concentrations from 10 μg/mL and resulted in over 90% of cell death. These results suggested that although the antimicrobial activity was identified against the main agents of bovine mastitis, the intramammary use of *T. minuta* oil may not be recommended. On the other hand, it is important to highlight the potential of the EO as an antiseptic or use as a sanitizer [36].

The data obtained in this study are similar to the data from the literature with regard to the efficiency of disinfectants based on medicinal plants against the microorganisms that cause mastitis. Nevertheless, there is only one study from Morão *et al*. [26] demonstrating the efficiency of the EO from *L. origanoides* in disinfecting teats.

The effectiveness of the experimental product presented in this study is further backed by the *in vitro* tests that displayed the good rates of inhibitory and bacteriostatic activity of the EO of *L. origanoides* [10,12,37,38]. It also verified the acute and chronic toxicity of this oil; the concentration of 120 μL/mL did not cause toxicity in mice [10].

## Conclusion

This study demonstrated that the experimental product based in *L. origanoides* EO did not influence the rates of the clinical and subclinical diagnoses with respect to isolated SCNs and *Streptococcus* spp. The disinfectant product obtained demonstrated no inferiority to the conventional product and did not alter the rates of IMI induced by SNC and *Streptococcus* spp. presenting the same rates of IMI in positive control. Hence, it can be concluded that this product acts like the disinfectant used in conventional protocols of pre- and post-milking. The disinfection of utensils allows for the minimizing of economic losses caused by mastitis and increases food security. The results of this study suggest that the formulated product with *L. origanoides* shows the potential to become an efficient alternative to conventional milking management and for the prevention of new IMI.

## Authors’ Contributions

NAM, RPM, MHFM, AAGF, and ACA designed the work. NAM, CNS, RPM, and LVMS collected the data and did the laboratory work. ACA and MHFM supervised the work. NAM, VAA, and ACA analyzed the data and drafted the manuscript. AREOX and MASX helped in critical review and data representation of the manuscript. All authors read and approved the final manuscript.
